# The Role of Soft Skills in Dental Education: Challenges and Importance

**DOI:** 10.1055/s-0044-1791938

**Published:** 2024-12-10

**Authors:** Murali Venkata Rama Mohan Kodali, Unati Sai Kodali, Srikanth Gadicherla, Komal Smriti, Anupam Singh, Zohaib Khurshid

**Affiliations:** 1 Department of Oral and Maxillofacial Surgery, College of Dentistry, King Faisal University, Al-Ahsa, Kingdom of Saudi Arabia; 2 Medical Graduate, Dr. NTR University of Health Sciences, Vijayawada, Andhra Pradesh-520008, India; 3 Department of Oral and Maxillofacial Surgery, Manipal College of Dental Sciences, Manipal, Manipal Academy of Higher Education (MAHE), Manipal, Karnataka, India; 4 Department of Oral Medicine and Radiology, Manipal College of Dental Sciences, Manipal, Manipal Academy of Higher Education (MAHE), Manipal, Karnataka, India; 5 Centre of Excellence for Regenerative Dentistry, Department of Anatomy, Faculty of Dentistry, Chulalongkorn University, Bangkok, Thailand; 6 Department of Prosthodontics and Dental Implantology, College of Dentistry, King Faisal University, Al Ahsa, Saudi Arabia

**Keywords:** dental education, problem-solving, professionalism, interpersonal skills

## Abstract

Soft skills encompass interpersonal abilities and values that enable individuals to adapt to diverse circumstances. In dentistry, a combination of soft and hard skills is crucial for successful practice and for achieving health care organization goals. However, dental schools face significant challenges in teaching and evaluating soft skills, including the subjective nature of assessment, variability in student engagement, and the lack of standardized curricula. The development of ethical and critical thinking skills is essential for students to balance competing interests in the profession while maintaining professionalism, such as dedication, accountability, competence, dependability, and respect for others. Health care professionals, including dentists, must cultivate soft skills to effectively guide and treat patients. This study aims to raise awareness of the importance of soft skills in dental education, specifically highlighting challenges in instruction and evaluation. Key conclusions include the need for a more structured approach to teaching soft skills, integrating them into the broader curriculum, and developing more objective assessment tools. By addressing these issues, dental education can better equip future dentists with the necessary skills for efficient patient care.

## Introduction

“The best teachers are those who show you where to look, but don't tell you what to see.”

–Alexandra K. Trenfor

To effectively perform their role in the community, graduates with professional degrees, including dentistry, must possess vital soft skills. The dental profession recognizes that clinical expertise, scientific knowledge, and interpersonal skills are vital components. Dental professionals who excel in interpersonal skills can listen to and communicate with their patients with empathy, allowing patients to understand their dental health and articulate their needs. 1 Since patients cannot objectively evaluate the quality of care provided, their relationship with the dentist becomes pivotal.

However, the importance of soft skills extends beyond interpersonal aptitude. Halley et al emphasize that in addition to clinical experience, dentists must display a pleasant demeanor and establish meaningful connections with patients, colleagues, and faculty members. 2 Although previous research on soft skills in dental training programs has focused primarily on specific components such as entrepreneurship, 3 professionalism, 4 5 6 7 8 9 critical thinking, 5 6 teamwork, 7 lifelong learning, 10 leadership, 8 and communication skills, 4 11 12 13 14 15 it is crucial to recognize that other soft talents are equally important.

Extensive research and expert consultation have identified various critical soft skills in the dental field, including communication skills, critical thinking and problem-solving, teamwork, leadership, information management and continuous learning, and entrepreneurship 16 17 ( Table 1 ). The development of these skills should begin during the training period to ensure their cultivation and integration into the professional practice of dentistry.

**Table 1:** Elements and subelements of soft skills

Elements	Sub elements
1. Communication skills	Nonverbal communication Communicate ideas in both speech and writing form with clarity, effectiveness, and assurance Having the capacity to improve interpersonal communication abilities Provide feedback and engage in active listening Communication skills with people from diverse cultures Communicate clearly, confidently, and at the listener's level Bargain and comes to a consensus Present using technology Using technology effectively in presentations
2. Problem-solving and critical thinking	See issues in ambiguous and complex situations, analyze, and reach fair judgments Comprehend and integrate into the community's and new workplace's culture Enhance thinking abilities, including the capacity to clarify, evaluate, and analyze conversations Focus intently on a job while being persistent Come up with concepts and alternate solutions Decide based on actual evidence Think creatively
3. Teamwork	Establish positive relationships, communicate successfully with others, and collaborate on shared goals Participate in the organization and coordination of the team's activities and take accountability for the group's choice Comprehend and switch between the duties of a group member and a team leader Acknowledge and appreciate other's attitudes and behaviors
4. Leadership	Evaluate and act when dealing with ethical dilemmas Behave morally, in addition to being societally accountable Possess a fundamental understanding of leadership theory The capacity for project management The capacity to comprehend and switch between the duties of a team member and a team leader Being able to manage team members
5. Management of information and ongoing education	Find and organize pertinent data from various sources Self-directed learning and the capacity to adopt new concepts
6. Entrepreneurship	Recognize commercial prospects Individual study Describe the corporate structure Create, seek, and seize opportunities for work

**Fig. 1:**
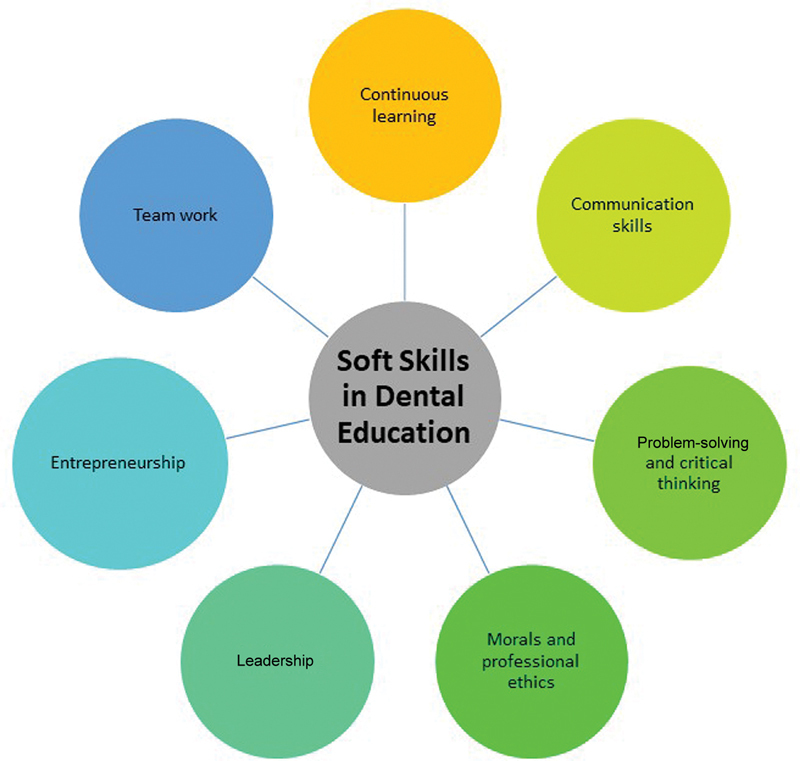
Soft skills in dental education.

Soft skills differentiate ordinary employees from future leaders. The success of a leader in guiding a team to complete a project relies on their interpersonal abilities. In addition, showing responsiveness and genuine care for patients shows a strong dedication to addressing their health issues and meeting their needs. The objectives of this review article are the following:

- To emphasize the importance of soft skills, their teaching and evaluation methods, and the challenges associated with their instruction and assessment.
- Examine how dental students apply their soft skills comprehensively and demonstrate their importance in health care ( Fig. 1 ).

## Communication Skills

Effective communication involves exchanging information, thoughts, and feelings through writing, speech, or other means. Interpersonal communication skills are crucial in health care professions, allowing skilled dentists to listen to patients, understand their needs, empathize, and conduct structured and professional consultations. 18 Communication problems with dentists often lead to patient complaints about dental appointments. 19 20 Effective communication can facilitate clinical diagnosis and decision-making and improve patient understanding. 18

## Problem-Solving and Critical Thinking

Modern dentists must excel at making diagnoses, formulating treatment plans, and providing optimal patient care. Critical thinking involves skillful and conscientious thinking that supports sound judgment by employing competence, knowledge, assumptions, and the ability to justify statements and actions. 5 6 The Commission on Dental Accreditation (CODA) in the United States has proposed new standards for teaching critical thinking and demonstrating this competency in comprehensive patient care. 6

## Teamwork

Teamwork is the combined effect of a group working efficiently and effectively. Individuals within an organization must cooperate as a team, assigning constructive roles to each team member. In dental care, teamwork is essential for efficient treatment delivery, with specialists playing different roles. Developing teamwork skills is vital to foster collaboration among dental team members. 7 21

## Leadership

Competent team leaders encourage participation, effectively distribute workloads, and bear the ultimate responsibility for the team's success. Effective leadership is necessary to advance a profession, adapt to change, find creative solutions, and build positive relationships with colleagues and patients. 8 22 A dental office can use effective business, management, and leadership skills to explore new opportunities and expand services. 23

## Continuous Learning

Being a lifelong learner is crucial for dentists to adapt to changing research paradigms. Relying on outdated knowledge is inadequate to meet professional expectations. Continuous learning encompasses practical and theoretical components that enable dentists to provide patient care aligned with professional standards. 24 25 Learning is vital for confidence and self-esteem, and dental schools must establish frameworks for continuous learning to prepare students for the future of dentistry.

## Entrepreneurship

Although dentists may not fit the traditional definition of entrepreneurs, possessing entrepreneurial skills can be valuable. Leadership, communication, flexibility, persistence, competition, inventiveness, originality, and personal traits contribute to entrepreneurial talents. Some or all of these skills are essential for dentists seeking to establish successful dental businesses. 3

## Discussion

Clinical professionalism encompasses the integration of learning and memory to effectively identify issues and engage in problem-solving within clinical interactions with patients. This includes demonstrating responsiveness to the socioeconomic, social, and political environment through skilled communication, critical analysis, professional competence, and information management. 26 Various educational methods have been employed to teach soft skills, such as presentations, workshops, case studies, project-oriented problem-based learning (POPBL), problem-based learning (PBL), and practical exercises in clinical skills and soft skills in laboratory settings. 12 27 28 The underlying principle is to provide a theoretical understanding of the skill before its practical application. However, within an already comprehensive curriculum, teaching and assessing soft skill competencies pose significant challenges for academic institutions.

Communication skills have been evaluated in the context of dental education. Yoshida et al conducted a study in 2001 to assess communication skills in 40 dental schools in the United States and Canada. 28 The findings revealed that only one-third of the schools offered group and interpersonal classes, with almost half of the institutions providing such courses primarily in the first 2 years. The subjects taught the most frequently included communication skills, patient interviews, patient education, and lecture. Didactic instruction and medical role-play with virtual patients were found to be the predominant methods of training communication skills. Practical communication training was provided prior to the start of clinical work. 27 The development of interpersonal skills was incorporated through the student instructors, including second-, third-, and fourth-year dental students and fourth-year medical students. 4 14 15 Students in their second year gained expertise in health care professional communication by observing dental faculty role-plays, which they then applied as third-year students when interacting with patients in the dental clinic. Positive evaluations were received from both students and peer teachers, who noted an improvement in communication abilities. Wagner et al investigated the effectiveness of teaching methods in improving medical and dental trainees' communication and counseling skills. 29 They found a correlation between online teaching and improved clinical abilities, suggesting further research to identify the types of students who might benefit most from this approach. Motivational interviewing (MI) has been considered an additional technique to enhance communication skills, and further research is needed to determine its efficacy in the dental setting. 30 Lucander et al conducted a trial workshop that uses an experiential approach and real-world scenarios to teach communication skills. 31 The workshop was practical for groups of 6 to 10 people, and the provision of systems to facilitate participant communication contributed to the positive response.

Sakaguchi modified the one-minute preceptor method that was developed, using iCARE an acronym for “inquire, Cultivate, Advise, Reinforce, and Empower, ” represents preceptor perspective that involve students' active engagement, participation, exploration, reflection, and implementation. 32 This strategy is believed to foster critical thinking among students. Critical thinking plays a crucial role in patient-centered care. 33 34 Identity, which involves self-awareness of how one's thoughts and actions can promote respect and empathy, is crucial. Practical communication skills improve patient satisfaction, treatment outcomes, and reduced anxiety. 33 However, there is a need to address the lack of clinical dental communication to enhance the learning and teaching of effective communication. Various factors must be considered when training communication skills, including the perception that dental professionals lack empathy. Empathy is related to effective communication and understanding of the patient's experience. Continuous personal skills training is suggested to be integrated into student education to counter the decreased compassion observed with increased patient exposure. 34 Holden argued that dental students might know what to say but struggle to express it effectively, potentially making patients feel patronized. 35 Chilcutt highlighted that communication skills training in dental schools focuses primarily on the dentist–patient interaction, while lacking the education in team leadership and communication necessary for dental practice preparation. 21

Problem-solving and critical thinking skills in dental education have been associated with clinical skills, diagnostic reasoning, and clinical soundness. 36 However, traditional lecture-based instruction has not yet been deemed effective for developing these soft skills. 5 6 Hendricson et al identified teaching approaches that have shown promise in fostering critical thinking. 36 These include engaging students in the analysis process, comparing different approaches, justifying the selection of a specific action plan, and documenting detailed analyses. Johnsen et al outlined three stages of critical thinking development. In the preclinical years, students are introduced to critical thinking principles, scientific papers, and evidence-based dentistry. 37 Students often look to their teachers as role models, emphasizing the importance of teachers who possess these skills for students to emulate. Benbelaïd et al found that dental students faced challenges in effectively outlaying treatment alternatives to patients, possibly due to lack of knowledge or difficulty applying their knowledge. 38 Dental schools may offer various clinical settings, such as dental clinics, primary health centers, or dental health clinics, as part of their educational approach. However, distinct divisions with separate evaluations can limit the integration of a comprehensive primary care theme.

There are different perspectives on how to promote critical thinking. DePaola et al argued that an environment of scientific inquiry fosters analytical reasoning in students. 39 At the same time, Chambers discovered that knowledge of research design, statistical procedures, and concepts from the dental literature could have improved critical thinking skills. Chambers also noted that reflective practice is sometimes mistaken for critical thinking and proposed that personality traits could predict critical thinking and clinical competence. 5 The quantification of critical thinking levels has presented challenges in the development of educational strategies to promote this skill. 36 Although critical thinking has been recognized as most assessed domain in dental education, the effectiveness of assessment procedures has not yet been evaluated. 19

Morals and professional ethics play a crucial role in dental education, to foster dentists' moral and practical responsibility. Field et al found that lectures and seminars were the primary methods used for ethics training in the United Kingdom. 40 The assessment of professionalism in dental schools often involves providing feedback to students after clinical interactions that could affect their professional growth and assigning letter grades. Integrating ethical considerations into PBL sessions, scenario presentations, small community presentations, and student reflections with standardized patients (SP) has been explored. These approaches do not aim to impose ethics on students, but rather to help them identify ethical dilemmas and make sound decisions. However, more research is needed to validate the effectiveness of these methods.

Dentists face ethical challenges daily and Brondani and Rossoff noted that distinguishing between clinical decision-making and ethical considerations can be challenging for dentists. 41 Fourth-year dental students at the University of Iowa identified ethical concerns related to patient limited resources, disagreements among specialists, clinical policies or procedures, and decisions made by patient surrogates. 42 Although students generally agreed that specific problems, such as adverse health outcomes and sexual assault, should be reported by dental hygienists, they were less inclined to report problems that could jeopardize their employment. Ethical decision-making skills are best achieved through first-hand experiences rather than simulations and lectures. 43 A survey evaluating ethics instruction in dental schools identified four areas of unmet needs: ethics to be comprehensively integrated across the curriculum including the clinical years, measuring and achieving ethics competence, staff development, and emphasis on teaching methods. 44

Teamwork and leadership play crucial roles in dentistry training, fostering an environment where clinical expertise and interpersonal skills converge. Teamwork enables dental professionals to collaborate effectively with other specialists, dental hygienists, nurses, and administrative staff to deliver comprehensive patient care. In dental schools, students are trained to work within teams, learning how to share information, communicate clearly, and coordinate roles to ensure treatment success. This collaborative learning environment reflects the interdependent nature of dental practice, where multiple team members contribute to patient outcomes. Studies show that teamwork enhances clinical decision-making and patient safety by reducing the chances of errors through cross-verification and shared responsibility. 45 Evans et al in their review suggested that the interprofessional education in dentistry is still in its infancy. Informal observations suggest that dental technology students who undergo an interprofessional curriculum are more equipped for collaborative practice. 7 Leisnert et al conducted a questionnaire-based study among the dental students and dental hygiene students for assessment of importance of teamwork. They concluded that promoting teamwork between dental students and dental hygiene students can enhance understanding of dental hygienists' skills and foster a comprehensive perspective on patient care and dental practice, effectively preparing both groups for their future careers. 46

Leadership, on the other hand, is vital for guiding dental teams and managing clinical practices. Dentists, often acting as team leaders, must make critical decisions, delegate tasks, and mentor less-experienced colleagues. Strong leadership in dentistry encourages continuous professional development and fosters a positive work culture that promotes patient-centered care. Moreover, dental education emphasizes leadership development to prepare future dentists to navigate challenges such as practice management, ethical dilemmas, and patient communication. Evidence indicates that leadership training improves problem-solving abilities and enhances the overall effectiveness of dental teams, promoting better outcomes in both patient care and workplace dynamics. 47 48

Organizations like National Health Service (NHS) actively promote and nurture future leadership in health care and dentistry. The NHS Long Term Plan emphasizes the critical role of strong clinical leadership in ensuring high-quality care within organizations and across the broader health care system. Dental professionals must take on leadership positions and represent the profession within the wider health care framework. 49 Some educational institutions like University of Southern California have a dedicated selective course on “Dean's Leadership Course,” which provides a compelling opportunity to nurture and cultivate leadership within university dental school. 50

Entrepreneurship in dental school plays a pivotal role in preparing students to navigate the business aspects of dental practice. Along with clinical skills, dental students must learn how to manage a practice, handle financial planning, and implement marketing strategies to ensure long-term success. Entrepreneurship education helps future dentists develop skills in risk management, innovation, and business decision-making, enabling them to establish and grow private practices or lead within dental organizations. By fostering an entrepreneurial mindset, dental schools equip graduates to adapt to market changes, adopt new technologies, and improve patient services, ensuring a sustainable practice. 51 52 Additionally, entrepreneurship encourages creative problem-solving and leadership, helping dentists identify opportunities for innovation in patient care and dental service delivery. As health care landscapes evolve, entrepreneurial dentists are more likely to embrace new treatment modalities, improve operational efficiencies, and contribute to community health initiatives. Integrating entrepreneurship into dental curricula thus ensures that students are not only competent clinicians but also savvy business owners capable of shaping the future of dental practice. 53 54

Singh and Purohit opined that dental enterprise encompasses dental schools, associations, dental industry, and various government agencies related to dentistry. Their review stated that the National Institute of Dental and Craniofacial Research (NIDCR) advocates for incorporating management skills, entrepreneurship, and technology transfer into dental education. 55 A study conducted by Santos et al examined the inclusion of entrepreneurship and marketing subjects in the curricula of Brazilian dental schools. Data from 563 active dental school curricula were analyzed, focusing on the presence of these subjects, their academic placement, and whether they were mandatory or optional. Results showed that only 22.6% of the schools included entrepreneurship subjects and 11.4% included marketing subjects, with no significant difference based on administrative systems. However, there was a notable difference in the inclusion of marketing subjects in the Southeast region of Brazil. The study concluded that Brazilian dental schools undervalue entrepreneurship and marketing education, highlighting the need for these subjects to be more prominently integrated to better prepare future dentists. 56

Lifelong education is vital in dental schools, establishing a framework for continuous learning and development. Various instructional tactics can foster competencies that support lifelong learning. An effective method is self-assessment. Redwood et al conducted seminars in which first-year dental students were encouraged to evaluate whether the standards and criteria used for clinical evaluation were being effectively taught. 57 Such a curriculum provides students with the necessary skills to pursue lifelong learning throughout their career. Polyzois et al emphasized the importance of dentists acquiring the expertise required for daily clinical practice and committing to lifelong learning as professionals. 58 Halley et al conducted a study to provide valuable information and advice to graduating dental students in the United States regarding the qualities dentists seek when selecting an associate. 2 They found that in addition to professional experience, positive demeanor and effective communication skills with patients, colleagues, and the dental team were essential attributes. Smithers et al highlighted the importance of incorporating personality traits into the selection process for dental students, regardless of academic achievement. 59 Dental educators face the ongoing challenge of developing appropriate evaluation techniques to assess the capabilities of graduating dental students, considering that evaluation should focus not only on individual skills but also on their integration. Evaluation in a clinical practice setting should focus primarily on qualitative measures that include rational reflection, ethical dilemmas, teamwork, management skills, and professional ethics, all of which are crucial to success.

**Fig. 2:**
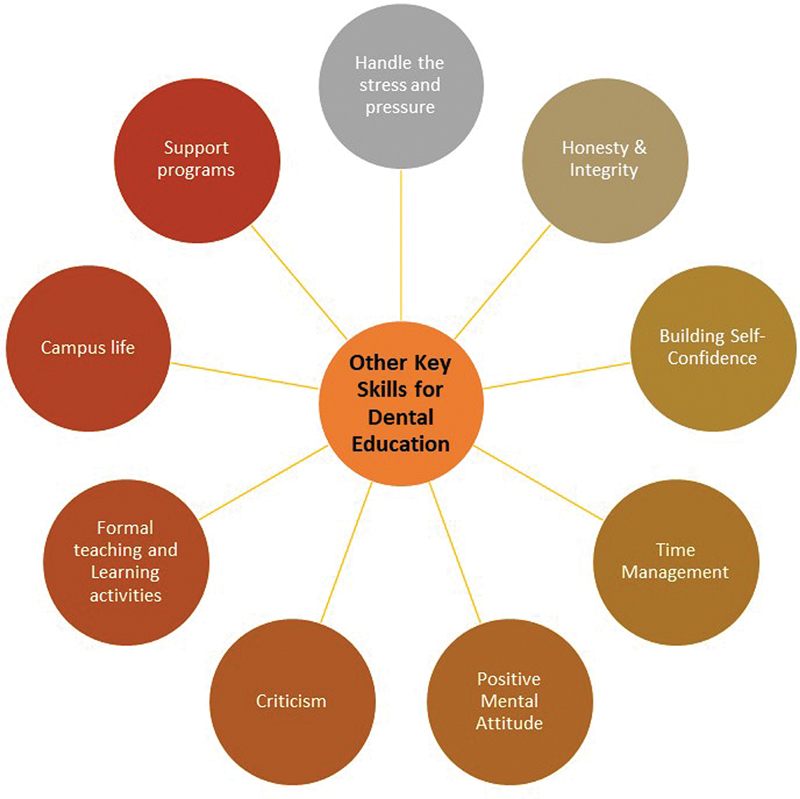
Other desirable key soft skills.

Some of the other key soft skills desirable during dental training are the following ( Fig. 2 ):

- **Handle the stress and pressure:** Individuals respond differently to high pressure levels. It is crucial to acquire effective stress management skills to ensure that pressure does not negatively impact performance.
- **Honesty and integrity:** Honesty and integrity are fundamental qualities of exceptional leaders on the job. Leaders must recognize that honesty and integrity are the cornerstone of effective leadership.
- **Positive mental attitude:** The impact of motivational and self-improvement books is often evaluated based on readers' proactive steps after reading them. In this regard, *Achievement through a Positive Psychological Attitude* stands out as one of the most popular self-help books of the 20th century. It has inspired countless individuals to pursue physical, mental, and moral well-being, as well as material success, while adhering to ethical principles and respecting the rights of others.
- **Time management:** Effective time management is a crucial soft skill that dentists must acquire to achieve desired results and goals in their professional activities. Dentists can overcome obstacles and meet tight deadlines by effectively managing their time. Good time management facilitates career advancement and improves professional reputation by allowing dentists to stay organized, meet deadlines, and effectively prioritize tasks.
- **Response to criticism:** Developing the ability to respond positively to criticism is a valuable life skill. One is likely to face criticism at some point, in a professional context or otherwise. How criticism is received depends entirely on one's response. Criticism can be used constructively to foster personal growth and improvement, or it can be handled negatively, leading to diminished self-esteem, increased stress, anger, and even hostility.
- **Building self-confidence:** Self-confidence refers to a positive belief in one's ability to navigate life and pursue personal goals. It entails having faith in one's skills and talents. Self-confident individuals are aware of their abilities and act accordingly, without relying on external validation for their sense of worth. They recognize their capacity and are willing to take advantage of opportunities that come their way. Self-confidence can have a self-fulfilling effect, as those lacking it may shy away from challenges or give up prematurely. At the same time, those who possess it are more likely to achieve success by leveraging their belief in themselves.
- **Enhancing soft skills through formal teaching and learning activities:** This approach incorporates standalone and integrated methodologies to cultivate soft skills. Integrated techniques are implemented through PBL, simulated laboratory courses, clinical sessions, School Dental Health Day, and community postings. Standalone strategies involve lectures in the Behavior Science module. A comprehensive assessment of soft skills is conducted before and after these sessions to gauge student progress. An interdisciplinary project serves as an example of combining standalone and embedded modules, where fourth-year students collaborate in pairs to undertake a self-directed research study under faculty guidance. Students must submit a handwritten research report for evaluation and present their findings orally or visually during the first quarter of their senior year. Completing the optional project is a prerequisite for graduating with a Bachelor of Dental Surgery degree.
- **Fostering soft skills through support programs:** Another approach is to establish various support programs within the dental school community. These programs aim to help faculty members and students develop their soft skills. An aspect of these programs involves inviting dental entrepreneurs to present their professional experiences and achievements, offering valuable insight into successful dental practice. Additionally, dental student associations actively promote extracurricular activities such as community involvement, social service initiatives, and sports competitions, fostering teamwork and communication skills among students. Furthermore, student exchange programs are facilitated between different dental schools, providing opportunities for cross-cultural learning and developing interpersonal skills. Although there is currently no formal evaluation of soft skills acquired through these support programs, it is anticipated that interactions and engagements within these initiatives will contribute to the improvement of soft skills among participants.
- **Acquiring soft skills through campus life:** The approach to achieve this involves fostering connections between dental students and learners in the supplementary curriculum. As part of this approach, first-year dental students must reside in university housing, providing them with opportunities for engagement and interaction. They are encouraged to actively participate in various activities organized by each residence hall, which include sports, arts, and charitable initiatives. Students are also encouraged to join community centers to expand their involvement further. Active participation in these campus events and organizations becomes a prerequisite for residency in the following academic year, emphasizing the importance of campus life in developing essential soft skills.

## Conclusion

In conclusion, integrating soft skills development into dental education is crucial to improving trainees' competency and improving dental health care. However, implementing effective soft skill instruction and evaluation requires a multifaceted approach. Further research can be conducted to understand dental schools' expectations regarding desired exit competencies, including soft skills. Exploring the perceptions of undergraduate dental students and teachers regarding the value of soft skills and their readiness to teach and assess these skills would be valuable. In addition, there is a need to establish appropriate evaluation techniques to measure the success of soft skill acquisition, as there currently needs to be a consensus on standardized assessment methods. Acquiring soft skills is essential for personal development, and there is ample room for future research on training dental students in soft skills and implementing a curriculum focused on developing these skills.
